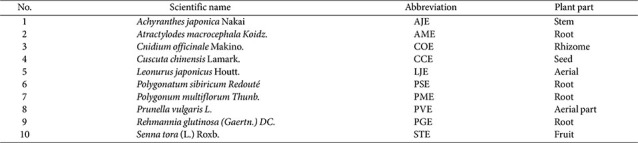# Leonurine-Standardized *Leonurus japonicus* Extract Promotes Recovery from Immobilization-Induced Muscle Atrophy via PI3K/Akt/mTOR Signaling in Mice

**DOI:** 10.4014/jmb.2604.04033

**Published:** 2026-06-01

**Authors:** Jiyeon Lee, Mi-Bo Kim, Changhee Kim, Chae Young Moon, Hyunju Kang, Jae-Kwan Hwang

**Affiliations:** 1 Graduate Program in Bioindustrial Engineering, Yonsei University, Seoul 03772, Republic of Korea; 2 Department of Food Science and Nutrition, Pukyong National University, Busan 48513, Republic of Korea; 3 Department of Biotechnology, College of Life Science and Biotechnology, Yonsei University, Seoul 03722, Republic of Korea; 4 Department of Food and Nutrition, Keimyung University, Daegu 42601, Republic of Korea; 5 School of Food Science and Biotechnology, Kyungpook National University, Daegu 41566, Republic of Korea; 6 Institute of Fermentation Biotechnology, Kyungpook National University, Daegu 41566, Republic of Korea

**Keywords:** Leonurine, *Leonurus japonicus*, Immobilization, Muscle atrophy, PI3K/Akt/mTOR pathway

## Abstract

Muscle atrophy involves the progressive loss of muscle mass and function, often linked to aging, disease, or inactivity. Although *Leonurus japonicus* and its active compound, leonurine, possess antioxidant and anti-inflammatory properties, their potential to improve muscle mass remains unknown. This study investigated the recovery-promoting effects and underlying mechanisms of a leonurine-standardized water extract of *L. japonicus* (LJW) and leonurine on muscle atrophy. We hypothesized that LJW and leonurine would promote recovery from muscle atrophy by activating the PI3K/Akt/mTOR pathway and suppressing FoxO3a-mediated proteolysis. Atrophy was induced in mice via 1-week hindlimb immobilization, followed by daily oral administration of LJW (150 or 300 mg/kg) or leonurine (30 mg/kg) for 1 week. LJW and leonurine not only improved exercise capacity and grip strength but also increased muscle volume, muscle mass, and cross-sectional area in the immobilized hindlimbs. At the molecular level, both treatments activated the mammalian target of rapamycin (mTOR) pathway for protein synthesis. They also increased forkhead box O3a (FoxO3a) phosphorylation and reduced FoxO3a-associated proteolytic markers, attenuating catabolic signaling by enhancing the phosphatidylinositol 3-kinase (PI3K)/Akt pathway. Furthermore, molecular docking analysis suggested that leonurine potentially interacts with the PI3K protein. Additionally, treatments reduced total nuclear factor kappa B (NF-κB) protein levels, which was accompanied by the downregulation of tumor necrosis factor α and interleukin 6. Taken together, LJW and leonurine enhanced recovery from immobilization-induced muscle atrophy, indicating their potential as novel therapeutic agents against muscle wasting.

## Introduction

Skeletal muscle accounts for approximately 50-75% of total body protein and plays a central role in locomotion and the regulation of various physiological processes [1]. Muscle atrophy is characterized by a decrease in muscle mass and function, arising from various conditions, including aging, chronic diseases, and disuse associated with prolonged bed rest, denervation, physical injury, and immobilization [2]. Furthermore, this loss of skeletal muscle is not limited to functional impairment and weakness but also contributes to metabolic disorders, such as diabetes and obesity [3]. Consequently, muscle atrophy markedly impairs quality of life and increases morbidity and mortality [4]. However, no pharmacological agents have been approved by the U.S. Food and Drug Administration for the treatment of muscle atrophy or sarcopenia, highlighting a significant unmet medical need for effective therapeutic strategies [5]. Therefore, safe and effective interventions targeting the underlying molecular mechanisms are required to restore muscle from atrophic conditions to its normal state.

Muscle atrophy is primarily driven by an imbalance between protein synthesis and degradation [6]. Inflammatory cytokines, such as tumor necrosis factor-α (TNF-α) and interleukin-6 (IL-6), are known to promote muscle wasting by enhancing protein degradation and suppressing protein synthesis. These cytokines act not only systemically but also locally in muscle tissue, thereby accelerating the progression of muscle atrophy [7, 8]. At the molecular level, the activation of forkhead box O3 (FoxO3a) plays a central role in mediating catabolic signaling, leading to the upregulation of muscle-specific ubiquitin E3 ligases, including muscle RING-finger protein-1 (MuRF1) and atrogin-1, which are key regulators of the ubiquitin–proteasome system [6, 9, 10]. In contrast, the phosphatidylinositol 3-kinase (PI3K)/ protein kinase B (Akt)/mammalian target of rapamycin (mTOR) pathway promotes protein synthesis and maintains muscle mass through the phosphorylation of downstream targets, such as 70 kDa ribosomal S6 kinase (p70S6K) and eukaryotic initiation factor 4E-binding protein 1 (4EBP1) [11, 12]. Therefore, targeting the balance between protein synthesis and degradation represents a promising therapeutic strategy for the treatment and recovery of muscle atrophy. In this context, plant extracts and phytochemicals have attracted considerable attention as potential therapeutic agents due to their diverse biological activities, including anti-inflammatory and anti-catabolic effects.

*Leonurus japonicus*, commonly referred to as the Chinese motherwort, is distributed in Korea, China, and Japan [13] and has traditionally been used as a medicinal and edible plant in East Asia. It is documented in classical medical texts for the treatment of gynecological disorders, including menoxenia, dysmenorrhea, and postpartum bleeding. Previous studies have reported that *L. japonicus* exhibits diverse bioactive properties, including antioxidant, anti-inflammatory, and anti-aging effects [13]. Leonurine (LEO), a major bioactive alkaloid isolated from *L. japonicus*, has also been reported to possess diverse pharmacological properties, such as anti-diabetic and anti-osteoporotic activities [14, 15]. In addition, both *L. japonicus* and LEO have demonstrated cardioprotective and neuroprotective effects [13, 14]. However, their potential recovery-promoting effects on skeletal muscle remain largely unexplored. In the present study, medicinal plants traditionally used in East Asian medicine were screened for their potential to regulate mTOR signaling. Among these, *L. japonicus* was selected for further investigation. Therefore, we aimed to evaluate the therapeutic and recovery effects of a LEO-standardized water extract of *L. japonicus* (LJW) and LEO on immobilization-induced muscle atrophy using both *in vitro* and *in vivo* models. In particular, we investigated the underlying molecular mechanisms associated with the regulation of protein synthesis and degradation, as well as the role of anti-inflammatory activity. To the best of our knowledge, this is the first study to demonstrate that *L. japonicus* promotes recovery from muscle atrophy through the modulation of protein turnover-related signaling pathways. We hypothesized that LJW and LEO would enhance recovery from muscle atrophy by activating the PI3K/Akt/mTOR pathway and suppressing FoxO3a-mediated catabolic signaling.

## Materials and Methods

### Preparation of Medicinal Plant Extracts

Dried medicinal plant materials were purchased from a traditional herbal market (Kyungdong Market, Republic of Korea) and authenticated by an expert based on their morphological characteristics. The medicinal plant materials used in this study are listed in Table 1. The selected medicinal plants are described in the traditional Korean medical text *Dongui Bogam* and have been traditionally used for conditions associated with musculoskeletal function, including muscle weakness, fatigue, and circulatory disorders. The dried plant materials were ground into a fine powder using a blender and extracted with 100% ethanol (v/v) at 50°C for 3 h using a shaking water bath (BS-20; Jeiobio Tech Co., Republic of Korea). Based on the screening results, selected samples were further extracted using 50% ethanol (v/v). The extracts were subsequently filtered, and the solvent was completely removed using a rotary vacuum evaporator (Heidolph Instruments GmbH & Co. KG., Germany) to obtain crude ethanol extracts. All extracts were dissolved in dimethyl sulfoxide and diluted to the desired concentrations prior to cell treatment.

### Cell Culture

L6 rat myoblasts were obtained from the American Type Culture Collection (ATCC, USA) and maintained in Dulbecco’s modified Eagle’s medium (DMEM; HyClone, USA) supplemented with 10% fetal bovine serum and 1% penicillin–streptomycin (Wisent, Canada). The cell line was authenticated by the supplier through morphological evaluation. Cells were cultured at 37°C in a humidified incubator with 5% CO_2_. For myogenic differentiation, cells were allowed to reach approximately 70-80% confluence, after which the growth medium was replaced with differentiation medium containing DMEM supplemented with 2% horse serum (Gibco, USA) and 1% penicillin–streptomycin. The differentiation medium was renewed every other day for 6 days to promote myotube formation. To establish an *in vitro* model of muscle atrophy, fully differentiated L6 myotubes were treated with recombinant rat TNF-α (50 ng/mL) for 12 h in the presence or absence of medicinal plant extracts or LEO (purity > 98%; Chemfaces, China), as indicated.

### Preparation of Standardized LJW

The dried aerial parts of *L. japonicus* were extracted with water (1:10 w/v) at 70°C for 4 h. After filtration to remove insoluble residues, the extract was concentrated using a rotary vacuum evaporator (Heidolph Instruments GmbH & Co. KG.) yielding 17.61%. The LEO content in *L. japonicus* extract was determined by high-performance liquid chromatography (HPLC, YL9100 HPLC system; Younglin Instruments Co., Ltd., Republic of Korea). Separation was achieved using a SunFire C18 column (150 × 4.6 mm, 5 μm i.d; Waters, USA). The mobile phase consisted of 1% phosphoric acid in water (A) and methanol (B). The gradient elution was programmed as follows: 95-80% (A) from 0 to 10 min, 80-65% (A) from 10 to 20 min, 65-50% (A) from 20 to 30 min. The flow rate was set at 1.0 mL/min and injection volume was 20 μL. Detection was performed at 277 nm. Standard solutions of LEO (Chemfaces) were injected to determine retention time and construct a calibration curve based on peak area. The LEO peak in the extract was identified by comparing retention times with the standard, and its content was quantified using the calibration curve. The LEO content in the LJW was determined to be 1.06% (w/w) (10.6 mg/g). The representative HPLC chromatogram is provided in Supplementary Fig. S1.

### mTOR Activity Assay

mTOR activity was determined using a mTOR sandwich enzyme-linked immunosorbent assay kit (Cell Signaling Technology, USA) according to the manufacturer’s instructions. Cells were lysed in NP-40 buffer containing a protease inhibitor cocktail, and protein concentrations were quantified using the Bradford assay. Cells were treated with each extract (10 μg/mL) for 12 h under basal conditions without any atrophy inducer. Parallel cell viability assays confirmed that these concentrations did not affect cell viability.

### Semi-Quantitative Reverse Transcription-Polymerase Chain Reaction (RT-PCR)

Total RNA was extracted from muscle tissues using TRIzol reagent (Takara Bio, Japan). RNA concentration and purity were determined using a NanoDrop 1000 spectrophotometer (Thermo Scientific, USA). Equal amounts of RNA were reverse-transcribed into cDNA using a reverse transcriptase premix (Elpis Biotech, Republic of Korea) with a GeneAmp PCR System 2700 (Applied Biosystems, USA). Semi-quantitative PCR amplification was performed using gene-specific primers (Bioneer, Republic of Korea) and SafeDry Taq PCR premix (CellSafe, Republic of Korea), as previously described [16]. The amplification conditions were as follows: denaturation at 94°C for 30 s, annealing at 58–60°C for 30 s, and extension at 72°C for 45 s for 25–30 cycles. PCR products were separated on 1.5% agarose gels and visualized using a G:BOX EF imaging system (Syngene). The number of PCR cycles was optimized to ensure amplification within the linear range. β-Actin was used as an internal reference gene. Densitometric analysis of the PCR bands was performed using ImageJ software. Band intensities were normalized to β-Actin and expressed as relative mRNA levels. Representative gel images are provided in Supplementary Figs. S2-S3.

### Animal Experiment

Eight-week-old male C57BL/6J mice were purchased from Daehan Biolink (Republic of Korea) and housed in an environmentally controlled facility (23 ± 2°C, 55 ± 10% humidity, 12 h light/dark cycle) at the Yonsei Laboratory Animal Research Center (Republic of Korea), with free access to a standard chow diet (Purina Rodent Chow 5001, LabDiet, USA) providing a physiological fuel value of 3.4 kcal/g, with calories derived from 28.9% protein, 13.6% fat, and 57.5% carbohydrates, and tap water. After a 1-week acclimation period, mice were randomly assigned to five groups (*n* = 6 per group): non-immobilized control (CON), immobilized control (IMA), IMA plus LJW treatment at 150 mg/kg/day (LJWL), IMA plus LJW treatment at 300 mg/kg/day (LJWH), and IMA plus Leo treatment at 30 mg/kg/day (LEO). Muscle atrophy was induced by immobilization using a previously described method [17]. Briefly, anesthesia was induced by intraperitoneal injection of tribromoethanol (325 mg/kg; Sigma-Aldrich). Except for the CON group, one tine of a staple was inserted into the gastrocnemius (GA) muscle of the right hindlimb and the other into the plantar surface of the foot using an F-35W skin stapler (Unidus, Republic of Korea). After 1 week of immobilization, the staples were removed, and the mice were orally administered by gavage for 7 days. During the administration period, the CON and IMA groups were treated with saline, whereas the treatment groups received LJW (150 or 300 mg/kg) or LEO (30 mg/kg). Following the treatment period, grip strength and exercise endurance were evaluated. The mice were then anesthetized with tribromoethanol (325 mg/kg, intraperitoneally) and euthanized by cardiac puncture. After confirming cessation of cardiac activity, the GA, soleus (SOL), tibialis anterior (TA), and extensor digitorum longus (EDL) muscles were collected from the right hindlimb and stored at −80°C until further analysis. All animal experimental protocols were reviewed and approved by the Institutional Animal Care and Use Committee of Yonsei University (approval number: IACUC-A-201906-923-03).

### Running Test

Exercise capacity was assessed using a motorized treadmill system (LE8710MTS; Panlab, Spain). Mice were subjected to a graded exercise protocol. The test began at a speed of 20 cm/s and was maintained for 20 min. Thereafter, the running speed was increased incrementally by 1 cm/s every minute until reaching a maximum speed of 35 cm/s. To ensure continuous running, a mild electrical stimulus (0.2 mA) was applied via the shock grid located at the rear of the treadmill. Exhaustion was defined as the inability of the mouse to resume running despite repeated electrical stimulation. At this point, the test was terminated, and total running time and distance were recorded for each mouse.

### Grip Strength Test

Forelimb and combined forelimb/hindlimb grip strengths were measured using a grip strength meter (Panlab). Each mouse was allowed to grasp a metal grid with its forelimbs or all limbs, and was then gently pulled backward by the tail with a constant force until the grip was released. The maximal force exerted was automatically recorded by the device. Each mouse underwent six consecutive trials, and the mean value of the measurements was used for statistical analysis.

### Micro-Computed Tomography (CT) Imaging

Mice were anesthetized with isoflurane, and the volume of the right hindlimb muscle was quantified using a dedicated small-animal positron emission tomography/computed tomography/single-photon emission computed tomography (PET/CT/SPECT) system (Inveon; Siemens, USA) at the Center for Evaluation of Biomaterials, Pohang Technopark (Republic of Korea).

### Histological Analysis

TA muscle tissues were fixed in 10% neutral-buffered formalin (Junsei, Japan), dehydrated, and embedded in paraffin. Paraffin-embedded tissues were sectioned and stained with hematoxylin and eosin (H&E). Histological images were obtained using an CK40 inverted microscope (Olympus, Japan) equipped with a T500 digital camera (eXcope, Republic of Korea) at a magnification of ×200. The cross-sectional area (CSA) of muscle fibers was quantified using ImageJ (National Institutes of Health, USA). Approximately 50-200 transversely sectioned fibers from 3-5 randomly selected fields per animal were analyzed. Obliquely sectioned fibers were strictly excluded from the analysis.

### Western Blot Assay

TA muscle tissues were lysed in NP-40 buffer (Elpis Biotech) containing protease inhibitors (Sigma-Aldrich) and centrifuged at 13,000 rpm for 10 min at 4°C. Protein concentrations were determined using the Bradford assay (Bio-Rad, USA). Equal amounts of protein were separated by SDS-PAGE and transferred onto nitrocellulose membranes (GE Healthcare, USA). Membranes were blocked with 5% skim milk and incubated with primary antibodies (1:1000) overnight at 4? Primary antibodies against PI3K (#4292), phosphorylated PI3K (p-PI3K; Tyr458/Tyr199; #4228), Akt; (#9272), phosphorylated Akt (p-Akt; Ser473; #4060), mTOR (#2972), phosphorylated mTOR (p-mTOR; Ser2448; #2971), p70S6K (#9202), phosphorylated p70S6K (p-p70S6K; Thr389; #9205), 4EBP1 (#9452), phosphorylated 4EBP1 (p-4EBP1; Thr37/46; #2855), FoxO3a (#2497), phosphorylated FoxO3a (p-FoxO3a; Ser253; #9464S), and α-Tubulin (#2144) were purchased from Cell Signaling Technology (USA). The primary antibody against nuclear factor kappa B (NF-κB p65; #sc-8008) was obtained from Santa Cruz Biotechnology (USA). Membranes were then incubated with horseradish peroxidase-conjugated secondary antibodies (1:5000; Bethyl Laboratories, USA). Protein signals were detected using an enhanced chemiluminescence (ECL) reagent (GE Healthcare) and captured using an imaging system (NFEC-2025-08-307766). Densitometric analysis of the protein bands was performed using ImageJ software. Target protein levels were normalized to α-tubulin, and phosphorylated proteins were normalized to their respective total protein levels.

### Molecular Docking

The crystal structure of PI3K (PDB ID: 8OW2) was retrieved from the RCSB Protein Data Bank [18, 19]. The structures of LEO (PubChem CID: 161464) and the protein were prepared using AutoDockTools 1.5.7 and PyMOL 3.1.0 as previously described [20]. Molecular docking was performed with AutoDock Vina within the PyRx platform (exhaustiveness = 9; grid center: 0.000, 2.000, -3.000; dimensions: 20 × 20 × 20 Å) [21]. Protein-ligand interactions were subsequently analyzed and visualized using PyMOL 3.1.0 (Schrödinger, USA) and PLIP [22].

### Statistical Analyses

All data are presented as the mean ± standard error of the mean (SEM). Statistical significance was defined as a *p*-value < 0.05. Comparisons among multiple groups were conducted using one-way analysis of variance (ANOVA), followed by Tukey’s post hoc test for multiple comparisons. Statistical analyses were performed using GraphPad Prism (version 10.0; GraphPad Software, USA).

## Results

### *L. japonicus* Extract Activates mTOR Signaling and Promotes Myogenic Gene Expression

**Fig. 1:**
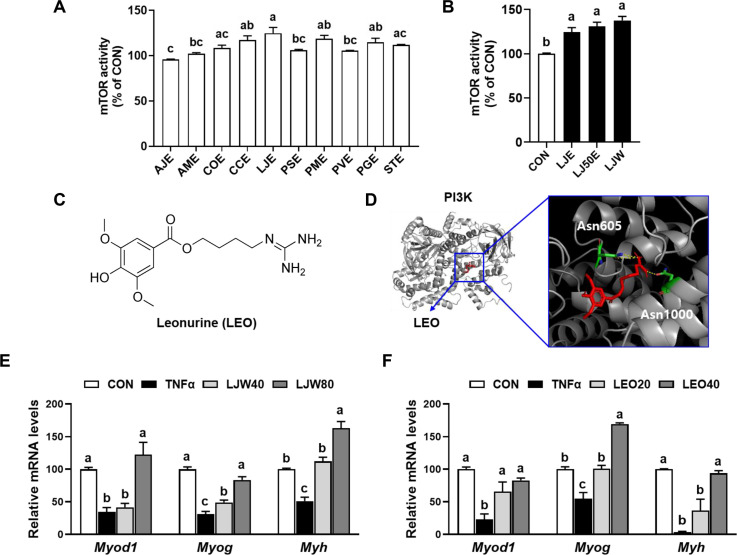
Effects of *Leonurus japonicus* extract and LEO on mTOR activation, molecular binding, and myogenic gene expression. (**A**) Screening of ten medicinal plant extracts for mTOR activity in L6 myotubes. (**B**) Comparison of mTOR activity among ethanol (LJE), 50% ethanol (L50E), and water (LJW) extracts of *L. japonicus*. (**C**) Chemical structure of LEO. (**D**) Molecular docking simulation of LEO (red) binding to the catalytic domain of PI3K (gray), displaying interactions with key residues (green). (**E**) Relative mRNA expression of myogenic regulatory factors in TNF-α–stimulated L6 myotubes with LJW; (**F**) with LEO. Data are presented as mean ± SEM. (*n* = 3-4 independent experiments). For mRNA expression, data were normalized to β-Actin. Bars not sharing a common letter denote statistically significant differences between groups (*p* < 0.05).

Ten medicinal plants documented in *Dongui Bogam*, traditionally associated with vitality and musculoskeletal health, were selected for screening. Their extracts were evaluated for effects on mTOR activity. Among them, the *L. japonicus* ethanol extract (LJE) exhibited the most pronounced activation, showing significantly higher activity than several other extracts, including *Achyranthes japonica* (AJE), *Atractylodes macrocephala* (AME), *Polygonatum sibiricum* (PSE), and *Prunella vulgaris* (PVE) (*p* < 0.05) (Fig. 1A). To further investigate solvent-dependent effects, LJE, 50% ethanol (L50E), and water (LJW) extracts were compared. All extracts significantly increased mTOR activity (*p* < 0.05), with the LJW showing the greatest effect (Fig. 1B).

Based on these findings, LJW was selected for subsequent analyses. The LEO (Fig. 1C) content in LJW was determined to be 1.06% (w/w). To explore a potential upstream molecular mechanism driving this mTOR activation, we hypothesized that LEO might interact with PI3K, a principal upstream kinase in the PI3K/Akt/mTOR anabolic pathway. Accordingly, molecular docking analysis was performed to evaluate the interaction between LEO and the catalytic domain of PI3K. The simulation revealed a stable binding conformation with favorable binding energy (-5.7 kcal/mol), indicating a potential interaction between LEO and PI3K (Fig. 1D). Notably, LEO formed interactions with key amino acid residues, including Asn 605, within the binding pocket. This suggests that LEO has the potential to interact with the PI3K structure, which may contribute to the modulation of the downstream Akt/mTOR signaling and promote recovery from muscle atrophy.

We next evaluated whether LJW and LEO influence myogenic regulatory factors under inflammatory conditions. TNF-α stimulation significantly suppressed the gene expression of myogenic differentiation 1 (*Myod1*), myogenin (*Myog*), and myosin heavy chain (*Myh*) compared to control cells. In contrast, LJW and LEO significantly prevented this suppression and partially restored the expression levels of these genes (Fig. 1E and 1F). Collectively, these findings suggest that LEO may contribute, at least in part, to the myogenic regulatory activity of LJW, potentially through mTOR-linked anabolic signaling under inflammatory stress.

### LJW and LEO Enhanced Recovery from Immobilization-Induced Muscle Atrophy and Functional Decline in Mice

**Fig. 2:**
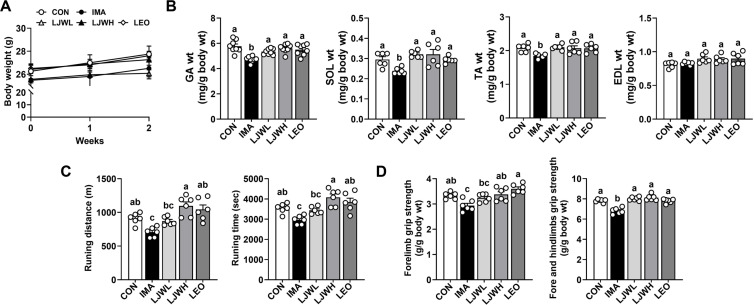
Effects of LJW and LEO on muscle mass and physical function in immobilization-induced mice. (**A**) Body weight changes during the 2-week experimental period. (**B**) Relative weights of the GA, SOL, TA, and EDL muscles normalized to body weight. (**C**) Running distance and running time assessed by a treadmill test. (**D**) Forelimb and combined forelimb/hindlimb grip strength. Data are presented as mean ± SEM (*n* = 6 per group). Bars not sharing a common letter denote statistically significant differences between groups (*p* < 0.05).

Given the effects of LJW and LEO on mTOR activation and myogenic gene expression in L6 myotubes, we next evaluated the effects of LJW and LEO on muscle recovery following immobilization in a mouse model of muscle atrophy. Body weights were comparable across all groups, indicating no systemic effects of immobilization or treatment (Fig. 2A). Hindlimb immobilization significantly reduced the weights of the GA, TA, and SOL muscles in the IMA group compared with the CON group. Treatment with LJW at both low (LJWL) and high (LJWH) doses significantly promoted the recovery of GA, TA, and SOL muscle weights, bringing them toward levels comparable to those in the CON group. Similarly, LEO administration significantly enhanced recovery from immobilization-induced muscle loss, increasing muscle weights across these muscles compared with the IMA group. In contrast, EDL muscle weight remained unchanged across all groups (Fig. 2B).

To determine whether the observed changes in muscle mass were accompanied by functional improvements, we next assessed exercise performance and grip strength in immobilization-induced mice. Hindlimb immobilization significantly reduced exercise distance and running time by 22.21% and 16.35%, respectively, compared with the CON group. In contrast, treatment with LJW and LEO improved exercise performance, with LJWH and LEO increasing exercise distance by up to 55.00 and 42.63%, respectively, whereas the LJWL group showed only modest effects (Fig. 2C). Consistent with the improved exercise capacity, grip strength was evaluated for both combined forelimb and hindlimb strength and forelimb strength alone. Both parameters were significantly reduced in the IMA group compared with the CON group, whereas LJW and LEO administration significantly improved grip strength (Fig. 2D).

### LJW and LEO Promoted the Recovery of Muscle Volume and Muscle Fiber CSA Following Immobilization

**Fig. 3:**
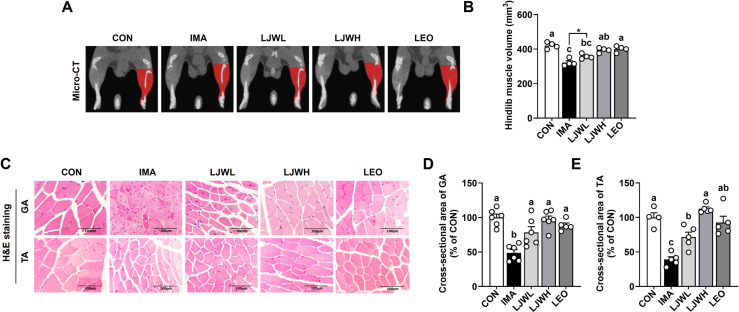
Effects of LJW and LEO on muscle volume and myofiber cross-sectional area. (**A**) Representative micro-CT images of the hindlimbs. (**B**) Quantification of hindlimb muscle volume. (**C**) Representative H&E-stained histological images of GA and TA muscle sections. (**D**) Relative cross-sectional area (CSA) of GA muscle fibers; (**E**) of TA muscle fibers. Data are presented as mean ± SEM (*n* = 4-6 per group). Bars not sharing a common letter denote statistically significant differences between groups (*p* < 0.05).

Consistent with the observed improvements in muscle mass and function, we next examined whether these effects were associated with changes in volume and muscle morphology. Micro-CT imaging revealed pronounced skeletal muscle atrophy in the IMA group, reflected by a substantial reduction in right hindlimb muscle volume compared with the CON group. LJW administration attenuated the immobilization-induced reduction in muscle volume in a dose-dependent manner, with the high-dose group (LJWH) restoring muscle volume toward control levels. Similarly, LEO administration significantly increased muscle volume relative to the IMA group (Fig. 3A and 3B).

To further assess whether these volumetric changes were accompanied by alterations in muscle fiber morphology, histological evaluation of skeletal muscle was conducted. In line with the micro-CT findings, the CSA of the GA and TA muscles were markedly reduced in the IMA group compared with the CON group. Notably, treatment with LJW and LEO significantly restored muscle fiber CSA relative to the IMA group (Fig. 3C-3E).

### LJW and LEO Suppressed Inflammation and Proteolytic Signaling in Immobilized TA Muscle

**Fig. 4:**
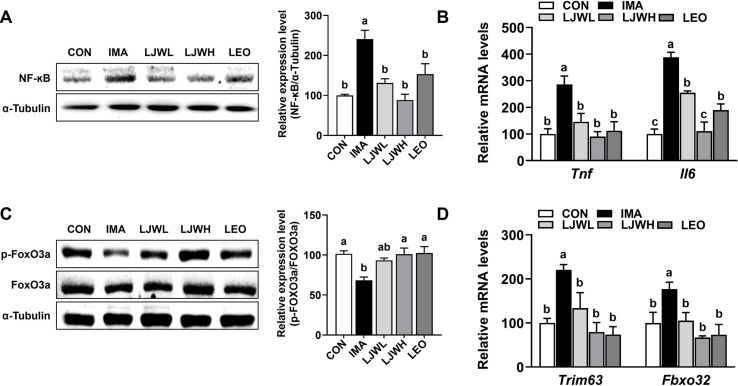
Effects of LJW and LEO on inflammatory signaling and proteolytic pathways in immobilized TA muscle. (**A**) Representative Western blot images and quantification of the relative protein expression levels of NF-κB. (**B**) The mRNA expression levels of pro-inflammatory cytokines, including *Tnf* and *Il6*. (**C**) Representative Western blot images and quantification of the relative protein expression levels of phosphorylated and total FoxO3a. (**D**) The mRNA expression levels of muscle-specific E3 ubiquitin ligases in TA muscle. Relative mRNA and total protein levels were normalized to β-Actin and α-Tubulin, respectively, whereas phosphorylated proteins were normalized to their corresponding total protein levels. Data are presented as mean ± SEM (*n* = 3 per group). Bars not sharing a common letter denote statistically significant differences between groups (*p* < 0.05).

To further elucidate the molecular mechanisms underlying the recovery-promoting effects of LJW and LEO against muscle atrophy, we investigated key signaling pathways associated with inflammation and protein degradation in TA muscle. Immobilization markedly increased the total protein expression of NF-κB compared with the CON group. In contrast, treatment with LJW and LEO effectively reduced the total expression levels of this inflammatory regulator (Fig. 4A). Consistent with increased NF-κB levels, immobilization induced a pronounced inflammatory response, as evidenced by significant upregulation of pro-inflammatory cytokines. The mRNA expression levels of *Il6* and *Tnf* were markedly elevated in the IMA group compared with the CON group. LJW administration dose-dependently reduced these increases, while LEO treatment also significantly suppressed cytokine expression (Fig. 4B).

Given the established link between inflammation and muscle protein degradation, we next examined FoxO3a signaling and its downstream targets. Immobilization significantly decreased the phosphorylation of FoxO3a, suggesting the activation of catabolic signaling. In contrast, LJW treatment dose-dependently restored FoxO3a phosphorylation, while LEO treatment also significantly increased p-FoxO3a levels (Fig. 4C), suggesting attenuation of FoxO3a-mediated catabolic signaling. Consistent with these findings, immobilization markedly upregulated the mRNA expression of muscle-specific ubiquitin ligases, such as *Trim63* (MuRF1) and *Fbxo32* (atrogin-1), compared with the CON group. However, both LJW and LEO treatments significantly downregulated the expression of these genes, indicating suppression of ubiquitin–proteasome–mediated proteolysis (Fig. 4D). Collectively, these results demonstrate that LJW and LEO enhance recovery from immobilization-induced muscle atrophy by attenuating inflammation associated with reduced total NF-κB levels and inhibiting FoxO3a-dependent proteolytic signaling pathways.

### LJW and LEO Restored Anabolic Signaling via the PI3K/Akt/mTOR Pathway in Immobilized TA Muscle

**Fig. 5:**
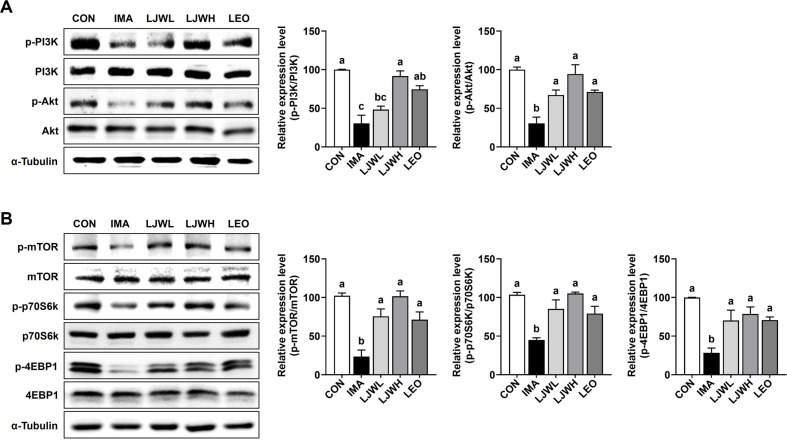
Effects of LJW and LEO on PI3K/Akt/mTOR anabolic signaling in immobilized TA muscle. (**A**) Representative Western blot images and quantification of the relative expression levels of phosphorylated and total PI3K and Akt. (**B**) Representative Western blot images and quantification of the relative expression levels of phosphorylated and total mTOR, p70S6K, and 4EBP1. Phosphorylated protein levels were normalized to their respective total protein levels, and total protein levels were normalized to α-Tubulin. Data are presented as mean ± SEM (*n* = 3 per group). Bars not sharing a common letter denote statistically significant differences between groups (*p* < 0.05).

To determine whether LJW and LEO modulate anabolic signaling involved in muscle protein synthesis, we examined the PI3K/Akt/mTOR pathway in TA muscle. Immobilization significantly decreased the phosphorylation levels of PI3K and Akt, indicating impaired anabolic signaling. In contrast, LJW treatment dose-dependently restored PI3K and Akt phosphorylation, while LEO treatment also significantly increased their activation levels (Fig. 5A).

Consistent with these findings, the phosphorylation of mTOR and its downstream targets, p70S6K and 4EBP1, was markedly decreased in the IMA group but significantly restored following LJW and LEO treatments (Fig. 5B). Collectively, these findings suggest that LJW and LEO promote recovery from immobilization-induced muscle atrophy by restoring PI3K/Akt/mTOR-mediated anabolic signaling.

## Discussion

This study provides evidence that LJW and its major bioactive compound, LEO, effectively promote recovery from immobilization-induced muscle atrophy. Our overall findings demonstrate that LJW and LEO effectively preserve skeletal muscle integrity by counteracting both structural and functional deterioration. The underlying recovery-promoting mechanism is driven by a dual-action molecular response: the suppression of pro-inflammatory cascades and the concurrent rebalancing of protein turnover through the activation of the PI3K/Akt/mTOR synthesis pathway and the attenuation of FoxO3a-mediated catabolic signaling. By elucidating these comprehensive mechanistic insights, this study establishes a pharmacological basis for utilizing LJW and LEO as novel therapeutic agents against muscle wasting.

The major features of muscle atrophy are the reduction in muscle function and muscle mass. Several causes of muscle wasting have been reported; for instance, chronic diseases, such as cancer, accelerate the breakdown of the balance between rates of protein degradation and synthesis owing to acute inflammation, resulting in muscle wasting [7]. Aging is another factor that causes muscle atrophy with chronic low-grade inflammation [23]. Disuse, which is represented by the instances of bed rest, space flight, and sedentary lifestyle, is a term used for muscle unloading [24]. In particular, disuse-induced muscle atrophy is a public issue in aging societies, since older individuals are associated with the bedridden state and fragility and they are easily exposed to muscle disuse [23]. However, treatments for relief of this condition are not available [25]. The persistent lack of FDA-approved pharmacological interventions for muscle atrophy underscores the urgent need for safe and effective natural-product-derived strategies that can enhance recovery by modulating complex muscle signaling networks.

In the current study, an animal model for immobilization-induced muscle atrophy representing disuse muscle atrophy was employed, as systemic alterations by circulating inflammatory cytokines through blood vessels can be ruled out in this model unlike sarcopenia or cachexia [7]. Consistent with previous studies [17, 26], immobilization significantly reduced the grip strength and mass of the hindlimb muscle, indicating that it effectively stimulates muscle wasting and atrophy. Importantly, oral administration of LJW and LEO successfully counteracted this immobilization-induced muscle wasting. LJW and LEO significantly restored muscle mass, volume, and CSA, while simultaneously improving actual muscle functions such as grip strength and exercise endurance in the immobilization-induced muscle atrophy model.

The robust *in vivo* efficacy observed in our model was achieved using specific dosages that were carefully selected based on previous pharmacological studies. Oral administration of *L. japonicus* at 200 mg/kg/day ameliorated nonalcoholic fatty liver disease [27]. *L. japonicus* treatment at 300 mg/kg inhibited posthemorrhagic chronic vasospasm and neovascularization [28]. Intragastric administration of LEO at 15 and 30 mg/kg/day reduced cardiac fibrosis through the NADPH oxidase 4/reactive oxygen species pathway [29]. Thus, in this study, the dosages of LJW were determined as 150 and 300 mg/kg/day and that of LEO as 30 mg/kg/day. These dosages correspond to human equivalent doses of approximately 12.2–24.4 mg/kg for LJW and 2.4 mg/kg for LEO based on body surface area normalization.

Having confirmed the phenotypic benefits of these optimal dosages, we next explored the underlying molecular mechanisms driving this muscle preservation. LJW and LEO have previously demonstrated therapeutic potential to attenuate inflammation-related diseases or symptoms [15, 30]. Thus, the anti-inflammatory properties of LJW and LEO were considered as one of the underlying molecular mechanisms by which they attenuated immobilization-induced muscle atrophy. Immobilization induced NF-κB protein expression and mRNA expression of *Tnf*, *Il6*, and *Ilb* in TA muscles [17, 26]. Similarly, the mRNA expression of *Tnf* and *Il6* and their major regulator, NF-κB protein expression was increased after immobilization treatment. *L. japonicus* attenuated production of TNF-α, IL-1β, and NF-κB-stimulated inducible nitric oxide synthase (iNOS) in lipopolysaccharide (LPS)-treated RAW264.7 cells [30]. LEO ameliorated IL-1β-induced inflammation in chondrocytes and murine osteoarthritis by reducing NF-κB signaling pathway and cyclooxygenase (COX)-2 and iNOS protein expression [15]. LEO also prevented cardiac fibrosis by downregulating iNOS, TNF-α, and NF-κB expression [29]. In the present study, by reducing total protein levels of NF-κB and subsequently downregulating pro-inflammatory cytokines like TNF-α and IL-6, LJW and LEO demonstrated an effective anti-inflammatory mechanism that mitigates muscle wasting. However, the effect on NF-κB activation remains to be clarified. Moreover, as pro-inflammatory cytokines like TNF-α are known to directly impair the PI3K/Akt signaling cascade and trigger FoxO3a activation, the anti-inflammatory action of LJW and LEO likely acts synergistically to restore muscle protein turnover.

Notably, our initial *in vitro* screening revealed a modest 20-30% increase in mTOR activity under basal, unstressed conditions. We consider this degree of activation to be biologically meaningful, as it indicates a safe 'anabolic priming' without causing cellular toxicity. The substantial phenotypic recovery observed *in vivo* is attributed not simply to this basal shift, but to the robust ability of LJW and LEO to restore the deeply suppressed PI3K/Akt/mTOR pathway under severe immobilization stress. While some mechanisms including inflammatory responses contribute to muscle atrophy, the other signaling cascades, typically the PI3K/Akt signaling pathway, were suppressed in the immobilized muscle. The insulin-like growth factor-1 (IGF-1) regulates muscle growth by upregulating the PI3K/Akt pathway [11]. In IGF-1 receptor knockout mice, the PI3K/Akt pathway was remarkably reduced, the number and area of myofibers were decreased, and insulin resistance developed [31]. In PI3K/Akt pathway-mediated muscle mass regulation, Akt stimulates the mTOR pathway, which mediates protein anabolism and prevents the activation of FoxO3a, a key regulator of proteolysis [11]. Although FoxO3a stimulates atrogin-1 and MuRF1, mitogen-activated protein kinase and NF-κB are other factors that increase the mRNA expression of atrogin-1 and MuRF1, respectively [32].

In accordance with this established signaling network, our results demonstrated that LJW and LEO successfully rebalanced muscle protein turnover by activating the PI3K/Akt/mTOR pathway to promote protein synthesis and concurrently suppressing the FoxO3a pathway to inhibit protein degradation. Specifically, LJW and LEO not only prevented atrogin-1 and MuRF1 expression by attenuating FoxO3a-mediated signaling but also stimulated the mTOR/p70S6K/4EBP1 pathway. In addition, LJW and LEO restored the PI3K/Akt pathway in the hindlimb immobilized mice. As Akt co-regulates mTOR and FoxO3a [11], these results indicate that LJW and LEO simultaneously regulate the Akt/mTOR and Akt/FoxO3a pathways in inhibiting immobilization-induced muscle atrophy. Crucially, our molecular docking simulation provided a structural rationale for this activation, revealing that LEO potentially interacts with the catalytic domain of PI3K by interacting with key residues such as Asn 605. This predicted structural interaction likely serves as a potential molecular modulator for the subsequent Akt/mTOR cascade activation.

While LEO serves as a primary driver of this PI3K activation, the comprehensive efficacy of LJW is likely augmented by its diverse phytochemical profile. Among the chemical constituents identified from *L. japonicus*, several compounds have been revealed to have potential for muscle atrophy in various muscle models. For example, β-sitosterol activated the PI3K/Akt pathway and increased muscle growth [33]. Caffeic acid stimulated formation of myotubes through activation of myogenic differentiation [34]. Quercetin inhibited unloading-induced disuse muscle atrophy through attenuation of ubiquitin ligases in mice [35]. Ferulic acid showed a hypertrophic effect on skeletal muscle of zebrafish through the activation of p70S6K and 4EBP1 [36]. Despite these promising findings, this study has a limitation in that it focused solely on an immobilization-induced model. Future studies exploring the pharmacokinetic profile of LEO in muscle tissue, as well as its efficacy in other atrophy models such as aging-induced sarcopenia, will be necessary to fully map its clinical applicability.

## Conclusion

In conclusion, this study demonstrated that LJW and its primary bioactive compound, LEO, effectively enhance recovery from immobilization-stimulated muscle atrophy in a post-immobilization recovery model. The synergistic effects of the diverse chemical constituents in LJW, combined with LEO's potential interaction with PI3K, highlight their strong therapeutic potential. Further clinical trials, including human toxicity and efficacy evaluations, are warranted to translate LJW and LEO into clinical applications for the treatment of muscle atrophy.

## Supplemental Materials

Supplementary data for this paper are available on-line only at http://jmb.or.kr.



## Figures and Tables

**Table 1:** List of medicinal plant materials and their abbreviations.