# Supplementary material for: Leonurine-Standardized Leonurus japonicus Extract Promotes Recovery from Immobilization-Induced Muscle Atrophy via PI3K/Akt/mTOR Signaling in Mice
Source: J Microbiol Biotechnol. 2026 Jun 1;36:e2604033. doi: 10.4014/jmb.2604.04033 (PMC13246295; doi:10.4014/jmb.2604.04033)
Supplement: Supplementary file 1 [file jmb-36-e2604033-supple.pdf]

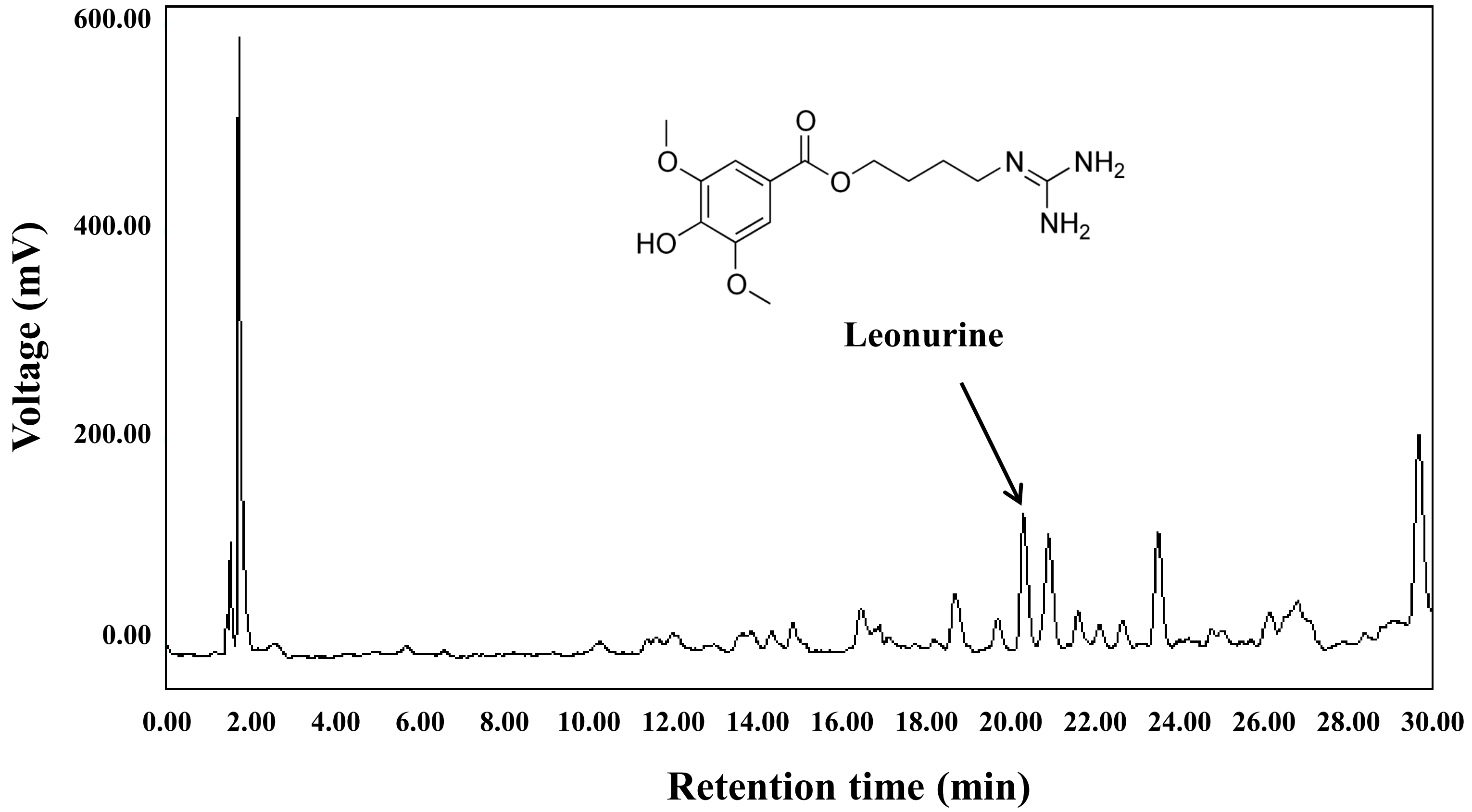

Supplementary Figure 1. High-performance liquid chromatography (HPLC) chromatogram of the *Leonurus japonicus* water extract (LJW). The representative chromatogram identifies the presence of leonurine in the extract. The specific retention time peak corresponding to leonurine is indicated by an arrow.

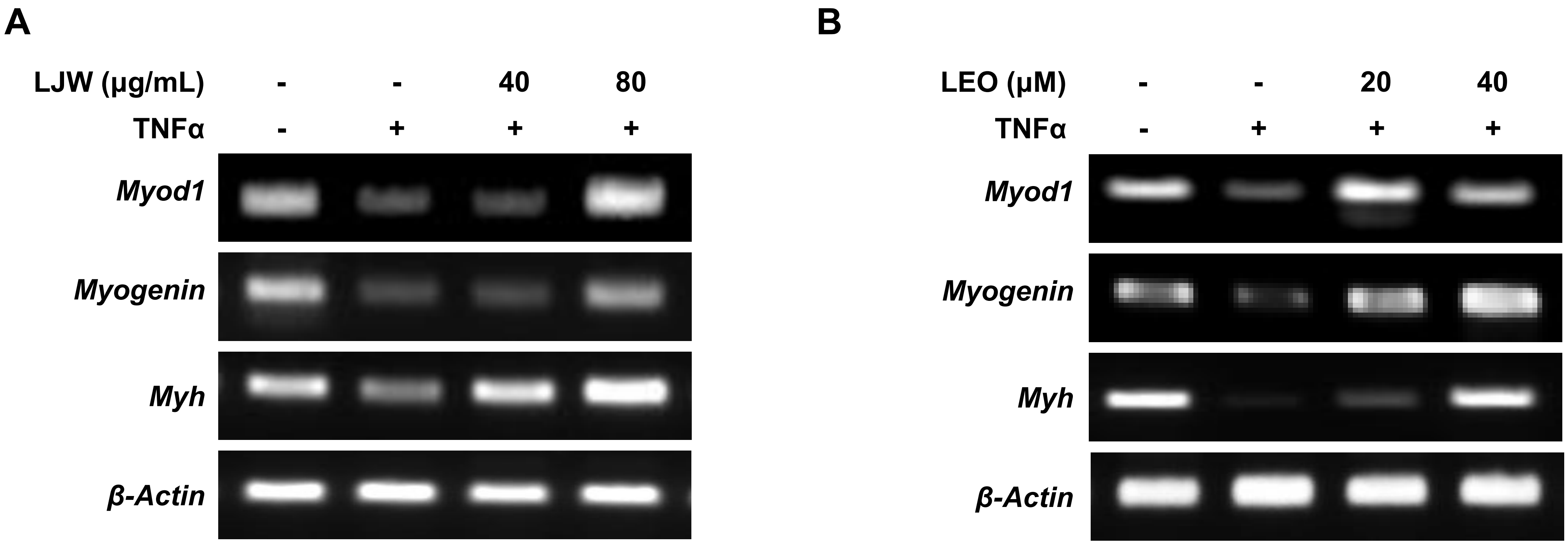

Supplementary Figure 2. Effects of LJW and leonurine on the mRNA expression of myogenic regulatory factors in TNFα-stimulated L6 myotubes. Representative gel images showing the mRNA levels of *Myod1*, *Myogenin*, and *Myh* in L6 myotubes stimulated with TNFα and treated with (**A**) LJW (40, 80 μg/mL) or (**B**) LEO (20, 40 μM). β-Actin was used as an internal control.

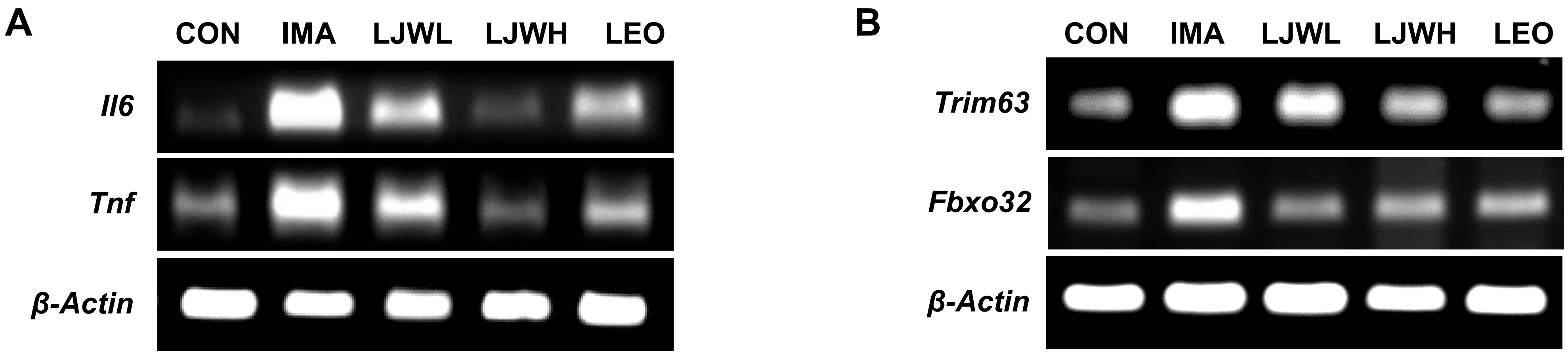

Supplementary Figure 3. Effects of LJW and leonurine on the mRNA expression of pro-inflammatory cytokines and muscle-specific E3 ubiquitin ligases in TA muscle. Representative gel images showing the mRNA levels of (**A**) pro-inflammatory cytokines (*Il6* and *Tnf*) and (**B**) muscle-specific E3 ubiquitin ligases (*Trim63* and *Fbxo32*) in the TA) muscle. β-Actin was used as an internal control for mRNA normalization. CON, normal control; IMA, immobilization-induced atrophy; LJWL, LJW 150 mg/kg; LJWH, LJW 300 mg/kg; LEO, leonurine 30 mg/kg.
